# DataCurator.jl: efficient, portable and reproducible validation, curation and transformation of large heterogeneous datasets using human-readable recipes compiled into machine-verifiable templates

**DOI:** 10.1093/bioadv/vbad068

**Published:** 2023-06-01

**Authors:** Ben Cardoen, Hanene Ben Yedder, Sieun Lee, Ivan Robert Nabi, Ghassan Hamarneh

**Affiliations:** Department of Computing Science, Simon Fraser University, 8888 University Dr W, Burnaby, British Columbia V5A1S6, Canada; Department of Computing Science, Simon Fraser University, 8888 University Dr W, Burnaby, British Columbia V5A1S6, Canada; Precision Imaging Beacon, University of Nottingham, Nottingham NG7 2RD, UK; Department of Mental Health and Clinical Neuroscience, University of Nottingham, Nottingham NG7 2UH, UK; Life Sciences Institute, University of British Columbia, Vancouver, British Columbia V6T 1Z3, Canada; School of Biomedical Engineering, University of British Columbia, Vancouver, British Columbia V6T 1Z3, Canada; Department of Computing Science, Simon Fraser University, 8888 University Dr W, Burnaby, British Columbia V5A1S6, Canada

## Abstract

Large-scale processing of heterogeneous datasets in interdisciplinary research often requires time-consuming manual data curation. Ambiguity in the data layout and preprocessing conventions can easily compromise reproducibility and scientific discovery, and even when detected, it requires time and effort to be corrected by domain experts. Poor data curation can also interrupt processing jobs on large computing clusters, causing frustration and delays. We introduce *DataCurator*, a portable software package that verifies arbitrarily complex datasets of mixed formats, working equally well on clusters as on local systems. Human-readable TOML recipes are converted into executable, machine-verifiable templates, enabling users to easily verify datasets using custom rules without writing code. Recipes can be used to transform and validate data, for pre- or post-processing, selection of data subsets, sampling and aggregation, such as summary statistics. Processing pipelines no longer need to be burdened by laborious data validation, with data curation and validation replaced by human and machine-verifiable recipes specifying rules and actions. Multithreaded execution ensures scalability on clusters, and existing Julia, R and Python libraries can be reused. *DataCurator* enables efficient remote workflows, offering integration with Slack and the ability to transfer curated data to clusters using OwnCloud and SCP. Code available at: https://github.com/bencardoen/DataCurator.jl.

## 1 Motivation

**Figure 1.**
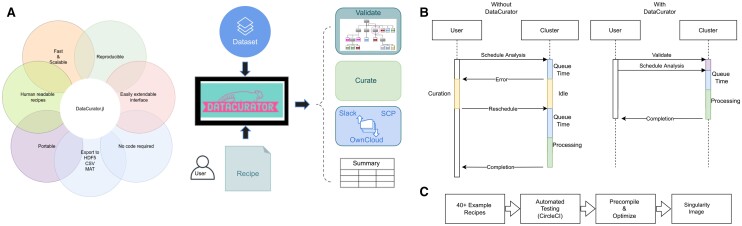
(**A**) A graphical overview of DataCurator’s key features. A user authors a recipe, without code, that is transformed by DataCurator into an executable template that can validate, curate and describe arbitrarily complex datasets. (**B**) A typical bioinformatics workflow deploys long computational analysis pipelines on large, often manually curated datasets. An error in data curation can be difficult to trace and leads to a rapid loss of productivity. Instead, with automated curation and validation using DataCurator, the loss of productivity is reduced to a minimum. (**C**) DataCurator’s functionality is tested automatically by running through more than 40 example recipes to verify its correctness. The test cases are then used by Julia’s precompilation routines to optimize, combining correctness with acceleration while self-documenting functionality. Once completed, the dependencies and optimized exectuable are stored in a singularity image for portable reproducibility

*DataCurator* came into being after the realization that our interdisciplinary research group was losing an avoidable yet significant amount of time on dataset curation and validation. Validation, in this context, is the process of verifying if a dataset, both in the hierarchical organization of files and directories, naming scheme, as well as the content of files, matches exactly what a user or algorithm expects. With modern computational pipelines processing terabyte-sized heterogeneous imaging datasets on shared computing clusters, where long compute jobs require even longer queue times, a single mistake or misunderstanding of data layout can require costly manual intervention and rescheduling, or even worse, produce inconsistent results hidden in the terabytes of output data. Communicating the exact data specification and layout between researchers acquiring the data and those developing algorithms based on the data is challenging and labour-intensive. Even if that communication and understanding are perfect, it is not machine-verifiable, so neither side can verify data at scale. In practice, extra validation and preprocessing (Famili *et al.*, 1997) codes are often added to pipelines as needed, but this does not solve the problem of unpredictable time and labour cost; at best, it prevents meaningless results and is usually specific to one use case. Furthermore, each interdisciplinary project run the risk of re-implementing ad hoc curation and validation code from scratch, either in the framework in which the pipeline is implemented or in cluster-specific shell scripts, neither of which are understandable by non-programmers. This problem is even more pressing for future users, be they adopters or reviewers, given that they were not involved in the creation of the datasets nor the development of the pipeline. *DataCurator* is intended to augment and complement, not replace, existing tools. FiJi (Schindelin *et al.*, 2012), for example, offers batch scripts and macros to make a reproducible pipeline, but these are specific to the platform and not interpretable by non-programmers. More recently, deep learning frameworks (Paszke *et al.*, 2017) allow for modular scripting for preprocessing before loading data into complex learning algorithms, and commercial offerings are taking this a step further with web-based pipelines. Automated validation tools are available for specific use cases [e.g. database migration (Paygude and Devale, 2013)] or automatically determining which subset of data causes machine learning algorithms to fail (Chung *et al.*, 2019). What is missing is a computing-language-agnostic method to validate and transform a wide range of datasets in a way that can be understood by data producers, reviewers, programmers, and machines alike, reducing ‘accidental complexity’ and letting scientists focus on ‘essential complexity’(Brooks and Kugler, 1987). Figure 1 illustrates at a high level what *DataCurator* offers. Figure 1B shows an example use case where the application of DataCurator can accelerate complex bioinformatics pipelines on computing clusters.

## 2 Human-readable recipes translated into machine-verifiable templates

**Figure 2.**
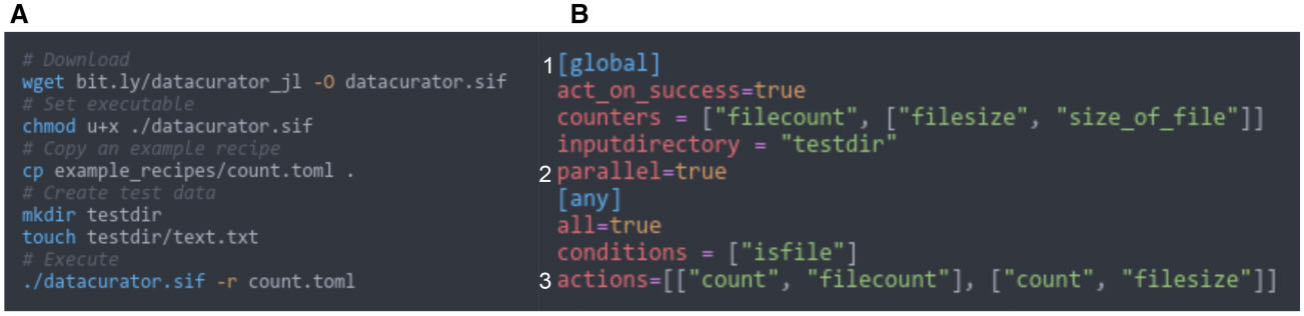
(**A**) The minimal instructions to get up and running with DataCurator on a cluster or a local machine using the Singularity image. Installing the singularity image of *DataCurator* can be completed with few lines only. (**B**) A minimal recipe that traverses a filesystem counting files and their respective sizes. A recipe is subdivided into a global (1) section configuring the properties of the template, such as parallelization, input directories and aggregation data structures, and a content (2) section that can optionally define hierarchical matching rules. In this case, the recipe will generate a template that can compute file counts and sizes of arbitrary data (3), at any scale, using all processors made available to it. On computing clusters, distributed filesystems are often quite slow to compute such results, so even this simple example can already save end users valuable time without relying on specific tools or expertise

*DataCurator* translates human-readable ‘recipes’, encoded in Tom’s Obvious Minimal Language (TOML) (Preston-Werner, 2018) into executable, scalable code, as illustrated in Figure 1. A recipe contains a global configuration section (Fig. 2B.1) and a template section with rules consisting of conditions (Fig. 2B.2) that trigger actions and/or counter-actions (Fig. 2B.3). Each TOML recipe, as the minimal example shown in Figure 2B, is translated into Julia (Bezanson *et al.*, 2017) code, which traverses heterogeneous datasets of files and folders of arbitrary depth, datatype and size.

**Figure 3.**
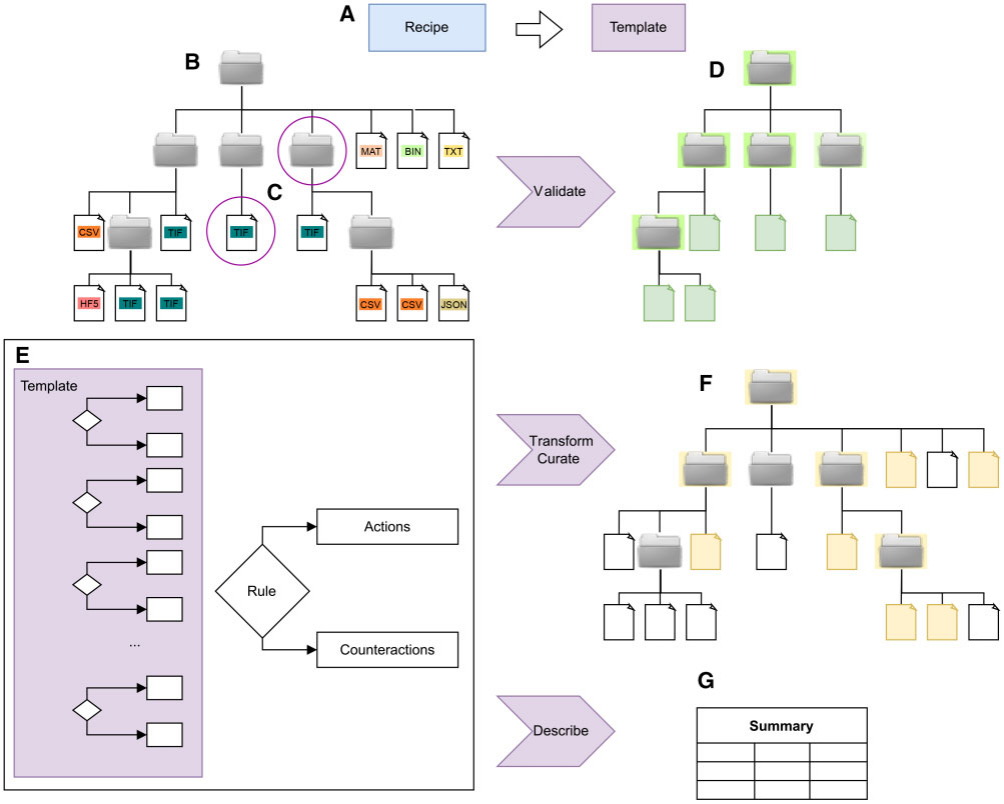
A human readable recipe is converted to a machine executable template (**A**). DataCurator processes a heterogeneous dataset (**B**). The template explores in parallel (**C**) folders or files to match predefined rules patterns from the recipe (**E**). The outcome can be validation (**D**), transformation (**F**), and summarizing of the matched data

## 3 Execution engine

*DataCurator* explores data in a top-down or bottom-up traversal. The aggregation engine, which collects files or parts of files based on user-defined filters, can combine any number of subsets of the traversed data using user-specified actions. A strongly typed execution engine combined with Julia’s multiple dispatches ensures recipes are translated into robust yet efficient code. Julia’s multiple dispatch (Muschevici *et al.*, 2008) system is relied on heavily to decode TOML recipes safely yet efficiently. Rules, which are condition-action-counteraction triplets, are evaluated from left to right in the order that they are specified. Optional counteractions allow reacting to both valid and invalid data. Explicit early exit actions are made available, as well as common actions such as annotated logging, filesystem manipulation and pattern matching.

## 4 Data type support

On top of standard file operations (copy, move, rename, delete, match), *DataCurator* can operate on the content of images, tables (CSV), HDF5, six single molecule localization microscopy formats (Cardoen, 2022), MAT, JSON, SQLite (file-based databases) and common mesh (geometry) formats and can be further extended to include new datatypes. While *DataCurator* examples currently focus on biomedical images, there are no technical restrictions to the kind of data it can process, with interfaces for processing files abstracted away to enable easy inclusion of new data type operations.

## 5 Rules and (counter) actions

Condition-action-counteraction triplets define how to validate data and, if found to be invalid, specify how to respond. A hierarchical context can be specified to ensure rules can only apply at a certain depth of the dataset, while repeated rules can be defined once in the global section for ease of reuse. Recipes are then self-documenting contracts between data producers and consumers, that can be both evaluated by humans and enforced by machines at scale. Figure 3 illustrates the pattern matching and capabilities in detail.

## 6 Composite rules

More complex conditions and rules can be composed by combining existing rules with logical operators. Composites themselves can be paired, meaning there is no limit to the expressiveness in templates. Handcrafted code, to achieve the same, would quickly blow up in complexity and become increasingly unmaintainable, let alone interpretable.

## 7 Machine-verifiable templates

Each TOML recipe is converted into equivalent Julia (Bezanson *et al.*, 2017) code. by a parser. Our parser resolves referenced actions and conditions into equivalent function calls. We heavily leverage Julia’s multiple dispatch system to realize this with the minimal amount of code yet retain maximal freedom of expression. For example, adding an action that processes a file is as simple as ensuring the named function is in scope.

## 8 Reusing existing libraries and scripts in Python, Julia and R

Using Julia’s R.jl, and Conda.jl packages enables the inclusion of any user defined functionality in the recipe in multiple languages, as long as the specified action or condition can be resolved to a callable function in that language.

## 9 Aggregation as a Filter-Map-Reduce engine

While individual file and folder processing is quite powerful, aggregation is frequently required where a set of files or folders is collected based on a set of conditions, combined, then transformed and saved. Example use cases include counting file sizes on a slow distributed file system where metadata can be prohibitively slow to retrieve, building sorted and preprocessed batch processing lists for scientific computing clusters, and all the way to stacking of 2D images into 3D volumes, or 3D over time into 4D. The aggregated image stacks can then be reduced by a maximum intensity projection, for example, then sorted per experiment or cell name from the metadata. DataCurator’s descriptive statistical capacity can be used for data validation. For example, segmentation output can be screened using DataCurator-generated per-slice object metrics and intensity distribution. The advantage of using *DataCurator* for these workflows is that it can do all of these operations in a single recipe using very simple instructions without requiring users to write code. In distributed computing terms, *DataCurator’*s aggregation engine supports the filter-map-reduce paradigm with user-defined actions. In addition to CSV, aggregation output can be saved in SQLite databases. A special case of aggregation is colocalization analysis of images, a workflow *DataCurator* fully supports with a range of metrics.

## 10 Lock-free multiprocessing

Fault-free parallel computing requires expertise that goes beyond the skill set of most programmers and data scientists. A single data race can easily compromise results, where all advantages of speed are nullified by the non-deterministic results. *DataCurator’*s multi-threaded engine uses thread-local data structures to ensure data races are impossible while maximizing throughput. As a result, there is no overhead from contention in locking shared data. The user simply needs to enable parallel processing in a recipe to reap the benefits.

## 11 Functionality

*DataCurator* is not intended to replace specialized image processing suites or database processing engines. *DataCurator* offers standard, scalable and interpretable execution of common, simple, repetitive, yet crucial workflows, such as filtering, computing statistics, masking, reducing or stacking, verifying type and dimensionality, slicing on composite conditions and aggregation of the outcome. Any operation that can be performed on a single image or table can be leveraged in the aggregation engine across multiple streams. It is possible to process an image multiple times, for example, to compute statistics on masked objects while also saving the parameters of its intensity distribution in selected slices.

## 12 Portability

Written in Julia, *DataCurator* can run wherever Julia does, i.e. on Linux, macOS, Windows, or BSD. However, to maximize deployment as well as reproducibility, we bundled *DataCurator* with all its dependencies into a single Singularity and Docker image. Existing R, Julia and Python libraries and functions can be used as-is by *DataCurator*, with documentation detailing how to incorporate them.

## 13 Documentation as test-driven performance

*DataCurator’*s functionality is demonstrated with more than 40 example recipes, which are in turn verified by automated tests for correctness. The execution profile of these tests is leveraged to intelligently precompile code and dependencies, ensuring maximum execution speed. The precompiled version and all dependencies are then bundled into a Singularity container for maximal reproducibility (Fig. 1C).

## 14 Remote support

To enable efficient remote workflow, integration with Slack is provided, as is remote transfer to OwnCloud and SCP-based systems. An example use case is to verify a recipe on local data, selectively copy subsets of the validated data to a cluster using SCP, and upload descriptive statistics to OwnCloud or Slack (Fig. 1A). At completion, a summary notification is then sent to a Slack workspace of choice. More advanced usage allows users to get Slack notifications as the curation proceeds, for example, to retrieve specific files in large datasets without having to log in to a cluster and wait until the results are completed.

## 15 Conclusion

*DataCurator* lets interdisciplinary research teams focus on scientific discovery by encoding data validation and transformation in human-readable, machine enforceable ‘recipes’, serving as self-documenting contracts between data producers and consumers. To enable efficient remote workflows, integration with Slack is provided, where *DataCurator* can report summary statistics on completion. In addition, user-based conditions allow *DataCurator* to message to Slack any parts of a dataset that match specific patterns. DataCurator supports remote transfer to ownCloud and SCP-based systems of both aggregate data and data matched by user-defined patterns. Finally, upon completion of the recipe, *DataCurator* can trigger the remote execution of large-scale compute jobs as well. *DataCurator* can remove complexity from existing pipelines and indirectly reduce accidental complexity, so researchers can focus on essential complexity. *DataCurator* is available under an open source licence (AGPLv3) at https://github.com/bencardoen/DataCurator.jl.

## Data Availability

No new data were generated or analysed in support of this research. Test data is available in the github repository.
